# Development of Sample-Adaptable Holders for Lightsheet Microscopy

**DOI:** 10.3389/fnana.2019.00026

**Published:** 2019-03-08

**Authors:** Thierry Laroche, Olivier Burri, Lalit Kumar Dubey, Arne Seitz

**Affiliations:** ^1^Bioimaging and Optics Platform, Faculty of Life Sciences, Ecole Polytechnique Fédérale de Lausanne, Lausanne, Switzerland; ^2^Faculty of Biology and Medicine, Department of Biochemistry, University of Lausanne, Lausanne, Switzerland

**Keywords:** lightsheet microscopy, sample preparation, clearing, custom, imaging, microscopy, fluorescence, lymph node

## Abstract

Multi-user core microscopy facilities are often faced with the challenge to adapt or modify existing instruments. This is essential in order to fulfill the requirements of the user community, who wants to image a wide range of model organisms with varying stains and sample thicknesses. In recent years, lightsheet microscopy has turned into an invaluable tool for both live and cleared sample imaging of many different specimens. This brought up new challenges in terms of sample mounting as the classical approach of attachment onto a coverslip cannot be universally applied. Here we describe the development of a diversified holder which extends the range of samples which can be imaged on a Zeiss Lightsheet microscope Z1. We focus on mounting strategies of cleared specimens; however, the holder and mounting strategy can be applied to live specimens too. The proposed methodology provides very high flexibility along with numerous possibilities for adaptation based on imaging specimen size, condition and available clearing reagents. Moreover, the described mounting strategies can be applied to other light sheet microscopes that can mount 1 mL syringes.

## Introduction

Selective Plane Illumination Microscopy (SPIM) or lightsheet microscopy (Huisken et al., 2004) turned into an invaluable tool in life sciences during the last decade (Girkin and Carvalho, 2018). In classical epi-fluorescence microscopy setups, the objective also fulfills the role of the condenser, i.e., it is also required to properly illuminate the sample. The key idea of lightsheet microscopy is to separate the excitation optical path from the emission optical path. A distinct set of lenses is used to illuminate the sample using a sheet of light, usually at 90 degrees with respect to the collection objective. This configuration provides three-dimensional (3D) optical slicing, allows for the use of cameras on the detection side and thereby greatly increases acquisition speed. In addition, this remarkably reduces phototoxicity compared to confocal microscopes while providing comparable optical slicing capabilities. Moreover, most lightsheet architectures allow samples to be viewed from various angles, which help compensate for optical aberrations inherent to large sample imaging (>100 μm tissue depth) (Preibisch et al., 2010).

However, the possibility to rotate the sample requires a different mounting strategy of the sample. In classical light microscopy, cells and smaller organisms are placed on a glass slide and mounted using coverslips. For lightsheet microscopy, this approach is less suitable. Various sample-mounting procedures have been published, and in most of the cases have been optimized for a specific specimen (Flood et al., 2013; Reynaud et al., 2014; Pampaloni et al., 2015).

The first commercial lightsheet microscopes were made available in 2012 and have now made their way into various microscopy core facilities across the globe. As core facilities have to cope with a variety of specimens, a flexible, versatile, and adaptable mounting strategy for lightsheet microscopy is envisaged.

We now present the development and optimization of a generic holder for the ZEISS Z1 lightsheet microscope. Our strategy was based on the following criteria: (1) It should be versatile for a large variety of specimens; (2) It should be quick and easy to assemble and not compromise image quality; (3) The components of the holder should be available world-wide for a reasonable price.

## Materials and Methods

### Mounting Strategy

By design, the Zeiss Z1 microscope can easily host samples up-to 4.5 mm. The classical sample mounting is based on a syringe which is inserted from the top into the microscope stage. The sample chamber is at that stage already installed and filled with the sample immersion media (e.g., water or buffer). The stage is used to move the sample relative to the objective and its diameter (designed to fit a 1 mL syringe) limits the sample size.

The release of a dedicated objective and chamber for cleared specimens triggered the need for this design choice to be circumvented, as cleared samples are easily larger than 4.5 mm in a given dimension (e.g., mouse brain, zebrafish, spinal cord, or lymph nodes).

Therefore, regardless of holder design or geometry, the sample mounting needs to be modified in order to mount and image larger specimens. The modified mounting protocol consist of three steps. First the sample holder is inserted into the stage. Contrary to the conventional mounting protocol, the imaging chamber is not inserted into the microscope at this timepoint. The sample then needs to be attached to the holder. This is a critical step as the sample is vulnerable to mechanical damages due to tensile forces as it freely hanging in the air at that stage. Once immersed into the liquid, buoyant force is reducing the tensile force which protects the sample. Finally, with the sample attached to the sample holder, the chamber can be inserted and the sample lowered into the medium for imaging.

The most delicate aspect of this mounting strategy is the time the sample spends suspended in the air before being lowered into the imaging chamber. This asks for an easy and rapid attachment step of the sample to the holder already inserted into the microscope.

### The Prototype

**FIGURE 1:**
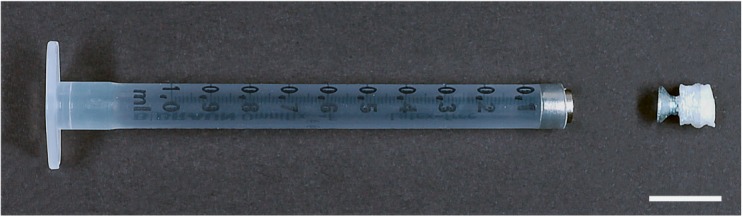
Image of a prototype to mount a large specimen. The tip of a 1 mL syringe (BD Biosciences) is cut off and a magnet is introduced. This can then hold a specimen attached with glue via a adapter, here a 3D printed PLA block attached to a flat head steel screw. Scale bar: 1 cm.

As the instrument is part of a multi user facility, it was desirable to provide a holder that could be used for a variety of specimen geometries. An initial prototype was based on a cut 1 mL syringe body (BD Biosciences) onto which a 4.5 mm magnet was attached (Figure 1). This magnet served to attract and hold a ferromagnetic piece. It allows for a quick conjunction of the sample and the holder, minimizing the danger of sample damage. As it is difficult to attach the sample directly to the ferromagnetic piece, the use of a spacer is recommended. This spacer can be designed based on the sample needs. In Figure 1, a 3D printed polylactic-acid (PLA) cube with a domed side was used. Onto one side the sample was glued using a cyanoacrylate-based compound. On the opposite side, a ferromagnetic screw was attached. This allows for a quick and reliable connection of the sample with the holder. We used a screw made of CCV-120 (Cr/V-alloyed, High Carbon Tool Steel). After a continuous usage of over 2 years, no remarkable signs of corrosion could be detected.

As mentioned above, the spacer can be created by 3D printing and its geometry can be shaped based on the sample’s requirements. Any ferromagnetic adapter could be used as long as the magnetic force is greater than the forces caused by gravity and the drag of the mounting medium.

However, this strategy came with some caveats. The short length of the adapter piece made positioning of the sample difficult, as it required tweezers in order to bring it close to the magnet. In some cases, the strength of the magnet would cause the adapter to snap once it was close enough. This caused shear forces that could detach the sample from the spacer. It is, however, possible to add a glued layer of 0.5–1 mm plastic or non-magnetized material (e.g., glass) on top of the magnet surface to damp the snapping effect. A more severe limitation of the holder was the difficulty to position the adapter onto the magnet precisely. As a result, the center of rotation of the sample could be different from the center of rotation of the stage. In addition, the fixed length of the adapter, involved cutting syringes different lengths in order to accommodate for samples of varying heights.

Nevertheless, the advantage of this prototype is that is can be easily rebuilt at low costs and with a minimum of effort. It can hold and mount large specimens. Due to the variable spacer, it can be used and adapted to almost any specimen compatible with the gluing compound.

### The Sliding Rod Holder

In order to overcome the limitations previously described, we developed and constructed a more refined holder so that (1) the snapping of the adapter would be avoided; (2) the positioning of the adapter would be more precise and reproducible and (3) the holder would have a variable length to easily accommodate taller samples.

**FIGURE 2:**
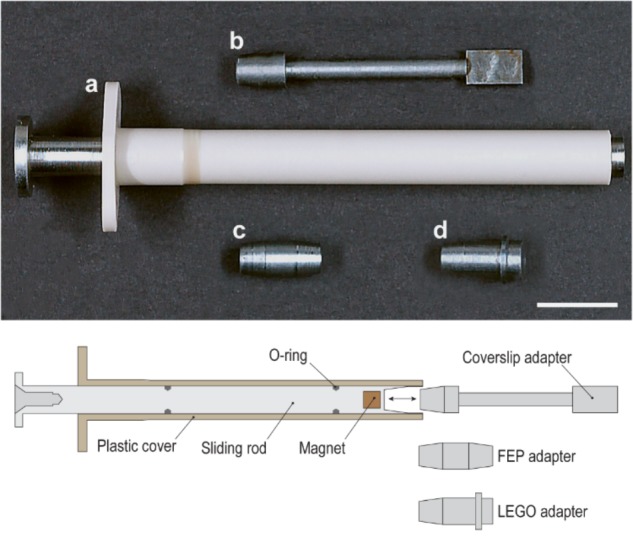
Photograph and schematic diagram of the sliding rod holder and its individual adapters: **(a)** plastic casing plus sliding steel rod, **(b)** coverslip adapter, **(c)** FEP adapter, **(d)** LEGO adapter. Scale bar: 1 cm.

The resulting holder, obtained from several rounds of optimization is summarized in the technical illustration shown in Figure 2 (bottom). The holder is made up of a hard-plastic cover (Figure 2a) (61.5 mm long and 7.0 mm in diameter) and a steel plunger (sliding rod) (73.5 mm long and 5.0 mm in diameter, CCV-120 Cr/V-alloyed, High Carbon Tool Steel) inserted into the cover. Unlike a typical syringe, two O-rings allow to easily position the steel plunger in the cover and hold its position, avoiding potential shear and movement of the piece.

At its end, the plunger has a tapered cavity containing a magnet that is slightly recessed. This allows us to modulate the strength of the magnet to avoid snapping. The cavity is built to receive a variety of adapters (Figure 2b–d), and the taper helps to gently and precisely position the adapters inside. All adapters are made of CCV-120 Cr/V-alloyed, High Carbon tool steel.

**FIGURE 3:**
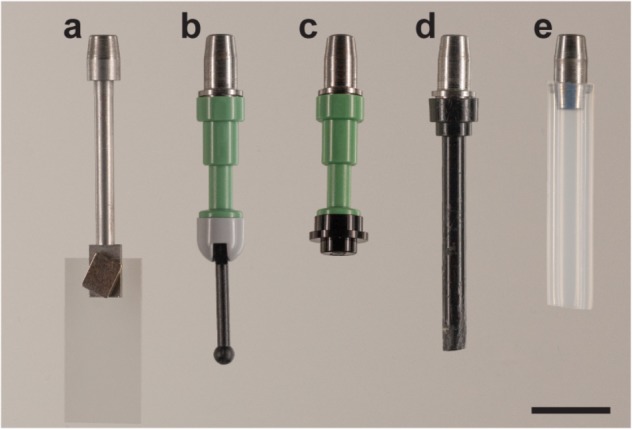
Adapters for the sliding rod holder. The versatility of the holder stems from the fact that different adapters can be used. Five different adapters are shown here: **(a)** cover slip adapter; LEGO adapters with various geometries, **(b)** LEGO Mini fig Telescope (64644) and LEGO Base with Black Lever (73587), **(c)** LEGO Mini fig Telescope (64644) and LEGO Round Plate 1 × 1 (4073/6141/15570); **(d)** LEGO Antenna 1 × 4 with Rounded Top (3957/15609/30064) and **(e)** FEP tube adapter with 4.0 × 6.0 mm FEP tube (Maagtechnic AG, ref. 10374302). Scale bar: 1 cm.

The first adapter (Figure 3a) introduced is a steel rod (35 mm long and 2.5 mm in diameter) with one flattened end 7.0 mm long, 4.0 mm wide, and 1.0 mm thick.

With this adapter, a coverslip is used to receive the sample. The coverslip itself – 22 mm × 10.5 mm Thermonox Plastic Cell Culture Coverslips (Thermo Scientific # 174934) – is attached with an additional magnet on the flat end of the steel rod. The sample can be attached with glue to the coverslip. This is advantageous in particular for soft samples as they can suffer from shear stress and deformations when moving through viscous clearing solutions like CUBIC (Susaki et al., 2015) or Histodenz^TM^ (Sigma-Aldrich -D2158-100G). Moreover, free hanging samples are difficult to image using multiview or tiled acquisitions due to drift and long sample setting times in viscous media. This drift is minimized when the sample is fixed to the coverslip. Thus, this approach turned out to be extremely useful when dealing with either large or soft tissues. The advantages and disadvantages of the proposed mounting strategy are summarized in Table 1.

**Table 1:** Advantages and disadvantages of the mounting strategy using the sliding rod holder with a coverslip.

Advantages	Disadvantages
Sample sizes of more than 4 mm in diameter	Sample can only be partially rotated.
Allows to image soft and delicate samples (e.g., spinal cord)	Sample can get in contact with the lens
Possibility of precise 3D acquisition	Glue makes the sample opaque
Possibility of tiled imaging	


The ability to image soft tissue reliably is clearly the main advantage of using the sliding rod holder. However, it comes with the disadvantage that specimen rotation is impaired, and the tissue might become opaque where it is in contact with the glue.

To circumvent this, we created another adapter (Figure 3b–d) that has a universal LEGO connector. LEGO pieces turned out to be extremely versatile when gluing specimens. They are available for a reasonable price, many different geometries are available, and they benefit from high chemical and temperature stability. The configuration shown in Figure 3b has proven useful to mount curved or twisted samples. The sample is glued to the tip of the lever and, using the hinge, positioned satisfactorily for imaging (at an angle). The hinge is then glued using cyanolit glue to avoid movement during imaging. Also all other variants shown have been successfully used in order to image cleared specimens.

The pros and cons for the LEGO adapter are summarized in Table 2. Note that with this adapter, we recommend to use only samples with a weight of less than 2 g. It is important to point out that other LEGO parts are also be compatible with the adapter and might serve to mount other samples as well.

**Table 2:** Advantages and disadvantages of the mounting strategy using the sliding rod holder with LEGO adaptors.

Advantages	Disadvantages
Sample sizes of more than 4 mm in diameter	Sample can get in contact with the lens
Allows to image samples longer than 25 mm	Glued part of the sample cannot be imaged
Possibility of precise 3D acquisition	
Possibility of tiled and multiview acquisition	


In addition, it is also possible to use the holder with a adapter capable of holding 6 mm fluorinated ethylene propylene (FEP) tubes as shown in Figure 3e. For mounting, tubes are filled with media, and sealed with a 2% or higher low melting-temperature agarose plug. The other end is then fixed with the metallic adapter. Small holes in the tube guarantee some gas and liquid exchange between the inside of the tube and the liquid of the incubation chamber, as previously described (Weber et al., 2014).

We have shown and described the adapters developed for the required applications of our users in the core facility. However, using the sliding rod holder, any adapter can be created, so long as it has a ferromagnetic element that can fit inside the holder.

The blueprints and specifications for the holder can be freely provided on demand.

### Sample Mounting Protocol

**FIGURE 4:**
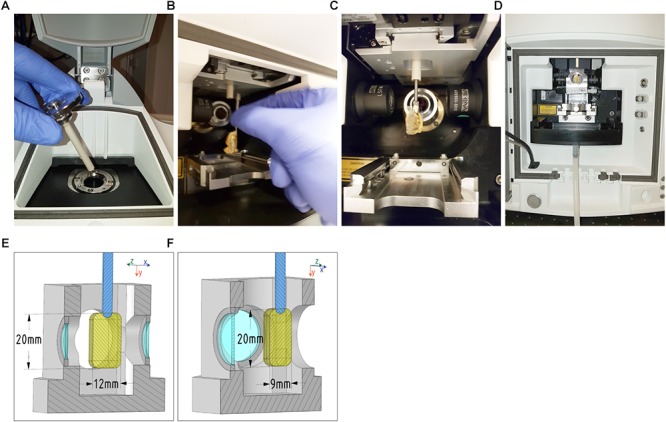
Mounting procedure and maximal sample dimensions for the Zeiss Lightsheet Z1. Top: **(A)** Insert the sliding rod holder onto the microscope stage, **(B)** then assemble the adapter (with sample attached) onto the sliding rod holder via the front door cavity. **(C)** Once in this position, **(D)** raise the stage as high as possible in order to insert the cell chamber and gently fill it with 25 mL of immersion liquid. Finally, lower the specimen into the immersion liquid of the cell chamber. Bottom: The maximum dimensions that can be acquired with a Zeiss Lightsheet Z1 are shown. The maximum height (22 mm) and length (12 mm) **(E)** of the sample are applicable to all chambers from Zeiss (Clearing chambers, water chambers, 5**×** objective chambers and standard chambers. The internal dimensions of the cell chamber are 20 mm (left-right) by 40 mm (top-down) by 24 mm (back-forth). The 9 mm maximum tissue thickness **(F)** is estimated from the working distance of the Zeiss Clr Plan-Neofluar 20x/1.0 Corr n.d. 1.45 objective.

The first step is to insert the sliding rod holder into the microscope stage (Figure 4A). Then, the sample can be connected via the adapter to the holder through the front cavity door (Figure 4B) and thus be assembled. Using the motorized z-drive of the instrument, or the sliding rod, the specimen is raised as high as possible to introduce the imaging chamber (Figure 4C). After introducing the chamber, 25 ml of immersion liquid are pumped in order to fill it. Finally, the specimen can be lowered into the immersion liquid of the imaging chamber (Figure 4D), ready for imaging.

With this mounting protocol, we are able to insert samples of a maximum width of 12 mm and a height of 20 mm (Figure 4E,F). The maximum imaging depth depends on the objective working distance, and can reach 5 mm using a Zeiss Clr Plan-Neofluar 20x/1.0 Corr n.d. = 1.45 with a working distance of 5.6 mm, with n.d. being the expected refractive index of the immersion media as well as the setting for the objective’s correction ring.

### Example Sample Application

**FIGURE 5:**
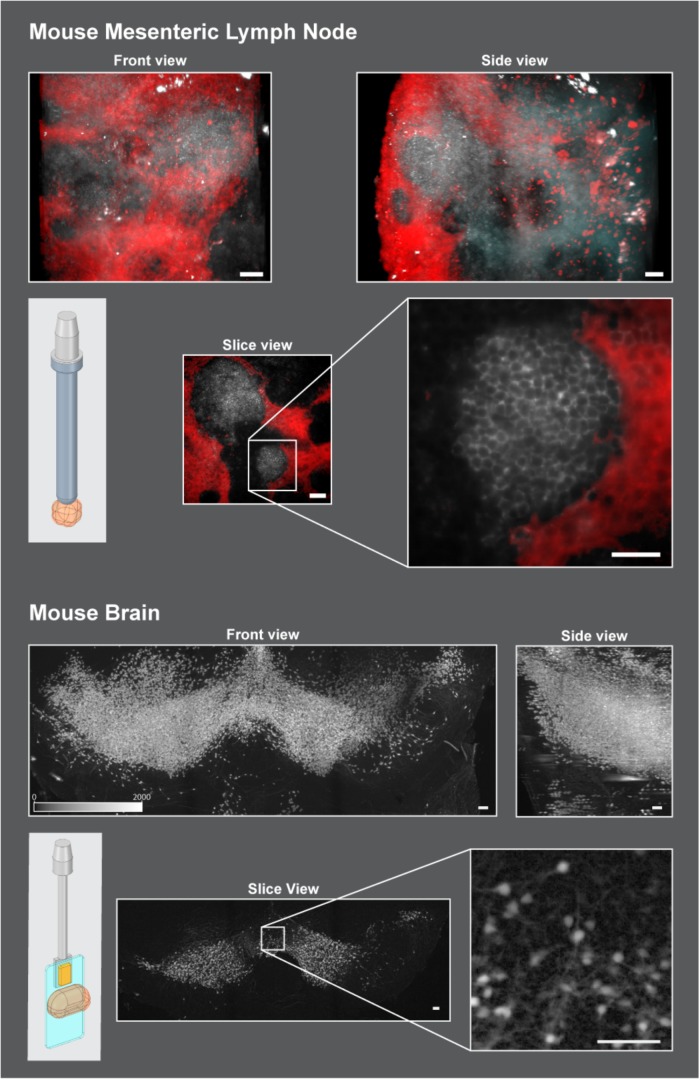
Mounting strategies in two use cases. Top: Cleared mouse mesenteric lymph node was glued onto a LEGO antenna and attached to the sliding rod holder with the LEGO adapter. Scale Bars = 50 um. TOP: Front views and side views of lymph node are shown to showcase the extents of width, height, and depth that can be acquired on this system with the sliding rod holder. The slice view represents a single slice roughly in the middle of the stack to show the resolution obtained. Bottom: Active Clarity Cleared mouse brain (Lee et al., 2016) was glued to a plastic coverslip and held in place onto the sliding rod holder with the slide adapter and a neodymium magnet (Figure 3b). 2 × 3 tiled acquisitions were performed to reconstruct the structure shown here. Scale bars = 100 um. To enhance tissue boundaries, images were gamma adjusted (gamma = 0.5). Schematic images on the top and bottom left of the figure show how the sample (orange) was mounted to work in the microscope using the developed adapters: top: LEGO adapter (Figure 3d), bottom: slide adapter (Figure 3a).

In order to demonstrate the versatility of the proposed sliding rod holder, two applications are shown in Figure 5. The first is a Mouse Mesenteric lymph node (Figure 5, Top), fixed overnight at 4°C in freshly prepared 1–2% paraformaldehyde in PBS, washed, and then processed for immunofluorescence staining for 3 to 5 days before imaging on a light sheet microscope. The thick sections were blocked with blocking buffer (PBS+1% BSA with 1% mouse and 4% donkey serum) overnight and stained for at 2 to 5 days with the primary antibodies (B220 and Lyve-1) followed by extensive washing in PBS (5x/1 h each) before incubation with fluorescently labeled secondary antibodies. After staining, samples were cleared as described previously using X-CLARITY Electrophoretic Tissue Clearing System (Logos Biosystems Inc.) (Dubey et al., 2017). After clearing, tissue was mounted using a LEGO antenna (LEGO Reference 3957/15609/30064) (Figure 5, schematic view, top and Figure 3d) and imaged using the Zeiss Lightsheet Z1 with the Clr Plan-Neofluar 20x/1.0 Corr n.d. = 1.45 objective in clearing solution with the LEGO adapter. Typical acquisitions are shown. Gray channel represents B cells stained using anti-B220 antibody, Red channel represents Lymphatics stained using anti Lyve-1 antibody. Scale bars = 50 um.

A second application shows the resulting acquisition of a Coronal stack of the midbrain of a P14 male mouse.(Figure 5, Bottom), where dopaminergic neurons were stained with tdTomato and cleared using active clarity protocol (Lee et al., 2016). Mounting was done using the slide adapter adapter (Figure 5, schematic view bottom and Figure 3a). A cut section of the brain was glued onto a plastic coverslip and then held to the adapter via a neodymium magnet. The size of the acquired stack is 4.8 mm × 1.8 mm × 1.6 mm and was reconstructed by acquiring multiple fields of view (3 in x and 2 in y) and stitching them together.

## Conclusion

We show the conception, construction and application of a new holder for the ZEISS light sheet microscope Z1 that can be beneficial across diverse samples. With the presented strategy, it is possible to mount and image a wider variety of cleared specimens with ease, and precise sample positioning. The holder does not compromise 3D imaging, sample rotation and supports tiled acquisition.

The modular design of the holder makes it a versatile tool for samples of varying scale and stiffness, and new mounting methods can be implemented into a new adapter easily. This is important in multi-user core facilities where adaptability is key to our users’ success. As the design follows the same external sizes of 1 mL syringes – as the ones used for the OpenSPIM (Pitrone et al., 2013) – all of its features are applicable far beyond the Zeiss Lightsheet Z1. With the increasing number of model organisms (Wolff et al., 2018) and imaging modalities in lightsheet, we are convinced the sliding rod holder will help various users across the field in imaging challenging samples with ease and encourage them to further develop novel mounting strategies.

## Data Availability

The datasets generated for this study are available on request to the corresponding author.

## Author Contributions

TL, OB, and AS designed the sliding rod holder and produced Figure 1–4. LD provided lymph node samples and performed the acquisition with TL, and produced the data used in Figure 5.

## Conflict of Interest Statement

The authors declare that the research was conducted in the absence of any commercial or financial relationships that could be construed as a potential conflict of interest.

## Glossary

**Sliding rod holder (holder):** the combination of plastic cover and steel plunger (sliding rod) with a tapered end inside which sits a magnet.

**Adapter:** ferromagnetic element that sits between the holder and the sample. The adapter has a beveled side that fits tightly with the holder’s tapered end. The other side has a variable geometry in order to accommodate elements adapted to the sample geometry.

**Sample:** the biological sample to be imaged. It is connected to the adapter via different strategies (e.g., plastic coverslips, LEGO pieces or FEP tubes).
